# Severe Pediatric Takayasu Arteritis Presenting With Cerebral Ischemia and Supra-Aortic Trunk Occlusion

**DOI:** 10.7759/cureus.96917

**Published:** 2025-11-15

**Authors:** Roy Ferrufino Mejia, Alicia Alejandra Peña-García, Adacrid Colunga-Duran, Johana Alejandra Roberto-Castillo, Gustavo Melo-Guzman

**Affiliations:** 1 Neurological Surgery, Juarez Hospital of Mexico, Mexico City, MEX; 2 Research and Academic Development, Saint Luke School of Medicine, Mexico City, MEX; 3 Neuroendovascular Therapy, Juarez Hospital of Mexico, Mexico City, MEX

**Keywords:** cerebral ischemia, large-vessel vasculitis, pediatric vasculitis, supra-aortic occlusion, takayasu arteritis

## Abstract

Takayasu arteritis (TAK) is a rare, chronic, autoimmune, and granulomatous vasculitis that predominantly affects large vessels, especially the aorta and its main branches, often leading to stenosis, occlusion, or aneurysm formation. Although TAK primarily affects young adult females, its occurrence in pediatric patients is uncommon and frequently associated with a more aggressive clinical course. We report a severe neurovascular presentation of TAK in a 16-year-old Mexican female patient who developed sudden-onset right hemiparesis, global aphasia, and multiple syncopal episodes. Marked interlimb blood pressure discrepancies were recorded, with upper limbs showing 63/30 mmHg and 66/35 mmHg compared with 127/47 mmHg and 135/52 mmHg in the lower limbs. Initial computed tomography revealed extensive cerebral ischemia involving the right posterior and left middle cerebral arteries. Diagnostic digital subtraction angiography (DSA) performed via a femoral 7-Fr introducer and JB2/pigtail catheters demonstrated complete occlusion of the supra-aortic trunks with collateral filling from intercostal arteries at T2-T4, consistent with type IIB TAK according to the Numano classification. Intra-arterial pressure measurements confirmed a systolic gradient exceeding 20 mmHg across the aortic arch. The patient required vasopressor support to maintain cerebral perfusion and received corticosteroids, anticoagulation, and neurocritical care. Clinical improvement was achieved with partial recovery of right-sided strength and language function. The absence of renal involvement and the coexistence of hidradenitis suppurativa raise potential questions about overlapping autoimmune mechanisms. This report emphasizes the neuroendovascular diagnostic approach and the importance of early vascular imaging and hemodynamic assessment in pediatric TAK presenting with cerebral ischemia.

## Introduction

Takayasu arteritis (TAK) is a chronic autoimmune granulomatous vasculitis that primarily involves the aorta and its major branches, producing progressive vessel wall thickening, luminal stenosis, thrombus formation, and, in advanced stages, aneurysm or dissection [1]. It ranks third among pediatric vasculitides after immunoglobulin A (IgA) vasculitis and Kawasaki disease, representing the leading large-vessel vasculitis of childhood [2]. Inflammation-driven vascular injury and downstream ischemia result in a broad clinical spectrum and significant morbidity if untreated. Early manifestations are often nonspecific, including fever, fatigue, weight loss, or myalgia, while disease progression leads to pulselessness, limb claudication, vascular bruits, and interlimb blood pressure discrepancies [3]. In children and adolescents, disease activity tends to be higher than in adults, following a more aggressive course with frequent complications such as renovascular hypertension, aortic regurgitation, and ischemic organ damage [4]. Neurovascular involvement, which may arise during active phases, represents one of the most severe forms of presentation and a major determinant of long-term outcomes [5]. Diagnosis integrates clinical, laboratory, and angiographic findings that demonstrate large-vessel narrowing or occlusion [6]. Although cytokine-targeted therapies have improved disease control, pediatric outcomes remain guarded because of high relapse rates and irreversible ischemic injury [7]. These challenges highlight the critical importance of early vascular imaging, timely recognition of neurovascular involvement, and coordinated multidisciplinary strategies aimed at preserving cerebral perfusion and preventing permanent neurological damage.

## Case presentation

A 16-year-old Mexican female patient with a history of prematurity (30 weeks' gestation) and untreated childhood asthma presented after an eight-month history of intermittent right upper limb paresthesia and weakness, as well as three self-limited syncopal episodes occurring in December 2024. She was admitted to the emergency department in February 2025 with sudden-onset right-sided weakness, loss of speech, fixed gaze episodes, and urinary incontinence, consistent with an acute cerebrovascular event. At presentation, her Glasgow Coma Scale score was 12 (E4V2M6). Neurological examination revealed motor aphasia, right homonymous hemianopia, right peripheral facial palsy, and cranial nerve involvement (right abducens and left oculomotor paresis). Muscle strength testing showed complete plegia of the right upper limb and moderate weakness of the right lower limb (0/5 and 3/5 on the Daniel scale, respectively) with normal strength on the left side (5/5). 

Blood pressure measurements demonstrated marked interlimb discrepancies, with upper limb readings of 63/30 mmHg (right) and 66/35 mmHg (left) compared with 127/47 mmHg (right) and 135/52 mmHg (left) in the lower extremities. Baseline laboratory testing revealed elevated inflammatory markers, mild cardiac enzyme elevation, and preserved renal function. Autoimmune and vasculitis-associated antibody panels, including antinuclear antibodies (ANA), antineutrophil cytoplasmic antibodies (ANCA), and antiphospholipid antibodies, were negative. Serial measurements over 13 days showed progressive normalization of erythrocyte sedimentation rate (ESR) and C-reactive protein (CRP) (Table 1).

**Table 1:** Inflammatory and biochemical markers during hospitalization ESR and CRP levels showed a progressive decline after the initiation of immunosuppressive therapy, reflecting reduced systemic inflammation. CK and serum Cr values gradually normalized, while mild hypoalbuminemia and anemia persisted. A transient decrease in Plt count was observed between days 3 and 10, with partial recovery at discharge. ESR: erythrocyte sedimentation rate; CRP: C-reactive protein; CK: creatine kinase; Cr: creatinine; Plt: platelet

Time point	ESR (mm/h)	CRP (mg/L)	CK (U/L)	Cr (mg/dL)	Albumin (g/dL)	Hb (g/dL)	Plt (×10³/µL)
Reference values	<15	0.5-3.0	30-200	0.6-1.3	3.5-5.0	12-16	150-400
Day 1 (admission)	62	18.4	890	1.64	3.6	13.3	182
Days 3-5	48	10.6	720	1.95	3.0	10.6	80
Days 7-10	32	4.8	483	1.30	2.7	8.7	76
Days 12-13 (discharge)	24	2.3	246	1.29	2.9	9.8	85

**Figure 1:**
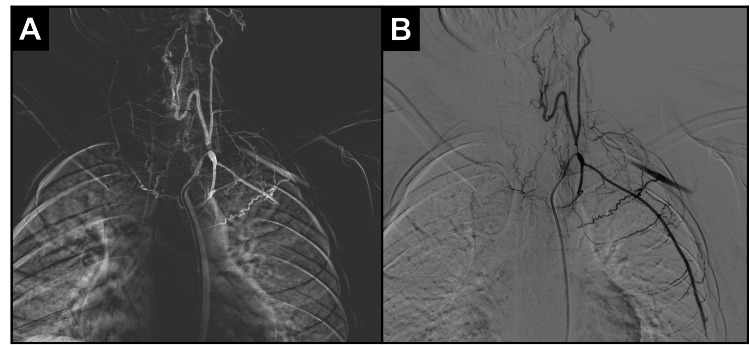
Digital subtraction angiography in AP Towne projection demonstrating supra-aortic occlusion (A) Absence of opacification in the brachiocephalic trunk and supra-aortic branches, with extensive collateral supply from intercostal arteries of the descending thoracic aorta. (B) Post-contrast image showing marked vascular tortuosity and retrograde filling through hypertrophied collateral networks compensating for the occluded supra-aortic circulation. AP: anteroposterior

Non-contrast brain computed tomography (CT) demonstrated chronic infarcts in the right posterior cerebral artery (PCA) territory and subacute ischemia in the left middle cerebral artery (MCA) distribution. Digital subtraction angiography (DSA) was performed via femoral access using a 7-Fr inguinal arterial introducer and a JB2 5-Fr diagnostic catheter mounted over a 0.035-inch hydrophilic guidewire. Sequential contrast injections from the ascending to the descending aorta revealed the complete absence of opacification of the supra-aortic trunks with extensive collateralization from intercostal branches of the descending thoracic aorta (Figure 1A-1B).

**Figure 2:**
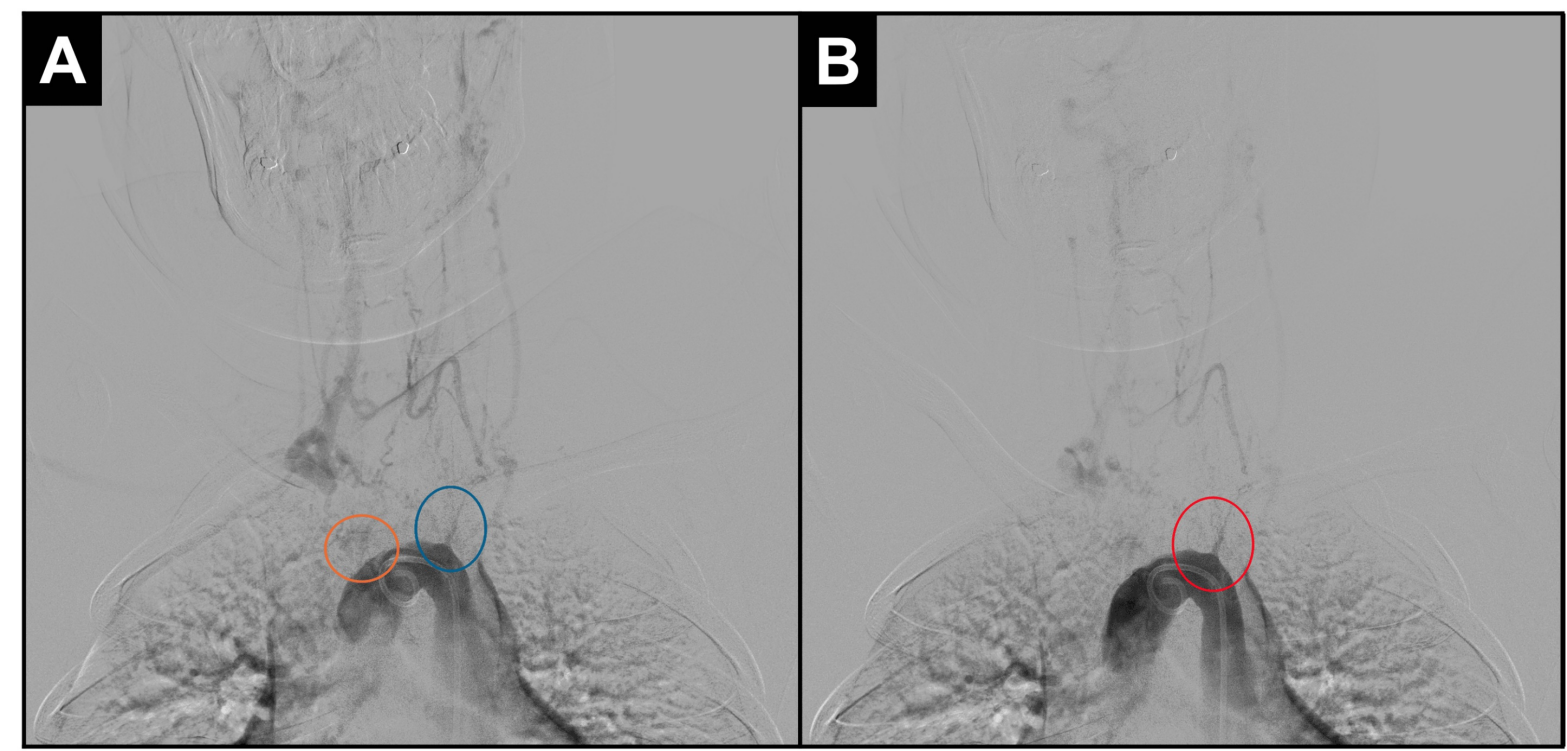
Selective aortic arch digital subtraction angiography showing the proximal supra-aortic occlusion (A) Contrast injection demonstrating absent opacification at the origins of the brachiocephalic trunk (orange circle) and left common carotid artery (blue circle). (B) Delayed phase revealing minimal distal opacification of the left common carotid artery through collateral retrograde circulation (red circle).

Selective aortic arch DSA confirmed the proximal occlusion of the brachiocephalic trunk and the left common carotid artery, with minimal distal filling through collateral circulation (Figure 2A-2B).

**Figure 3:**
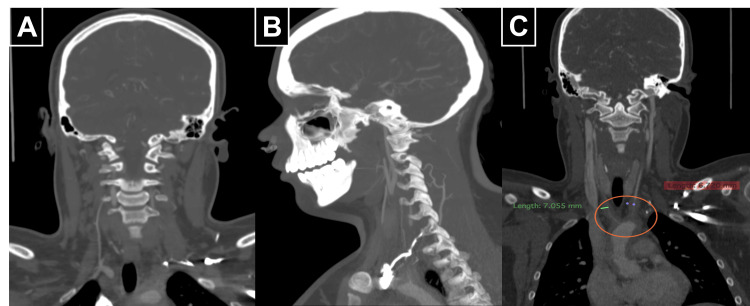
Contrast-enhanced computed tomography showing wall thickening and the truncation of supra-aortic vessels (A) Coronal view showing the abrupt truncation of the great vessels with loss of opacification beyond the aortic arch. (B) Sagittal reconstruction revealing the concentric inflammatory wall thickening of the ascending aorta and cervical arteries. (C) Coronal view with multiplanar measurements confirming the mural thickening and near-complete occlusion of the brachiocephalic trunk and left common carotid artery (orange circle).

These findings correlated with contrast-enhanced CT angiography, which showed circumferential wall thickening and abrupt truncation of the great vessels beyond the aortic arch (Figure 3A-3C) and explained the previously reported absence of luminal opacification, reflecting complete hemodynamic occlusion rather than isolated mural thickening.

**Figure 4:**
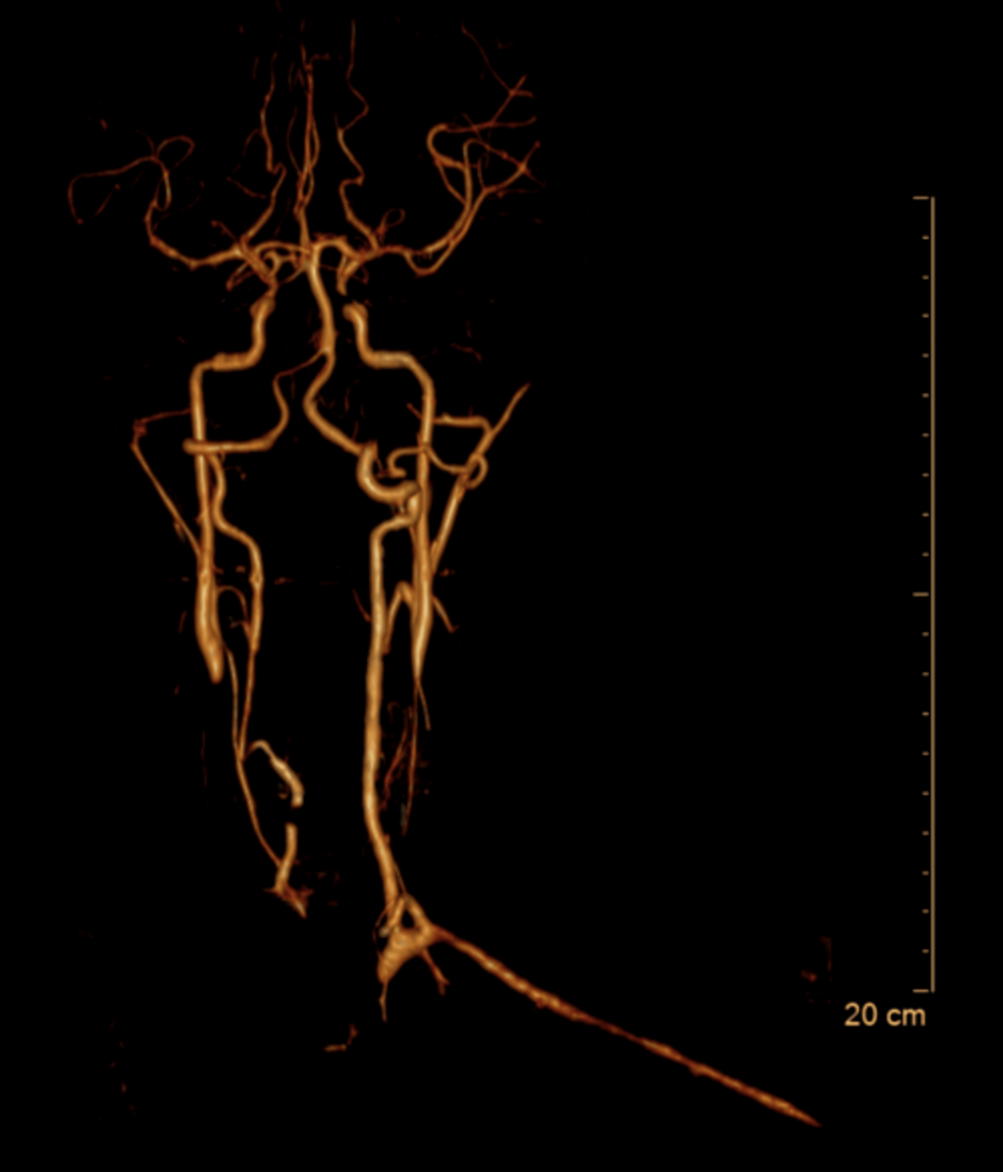
Three-dimensional computed tomography angiography revealing vascular truncation and tortuosity Three-dimensional reconstruction showing the marked truncation and pronounced tortuosity of the supra-aortic vessels, particularly involving the brachiocephalic trunk and carotid arteries, with relative preservation of distal intracranial circulation.

Three-dimensional reconstruction demonstrated tortuosity of the supra-aortic branches, with preserved distal intracranial circulation (Figure 4).

Intra-arterial pressure monitoring revealed a systolic gradient of 25 mmHg between the ascending aorta and distal arch, confirming hemodynamic obstruction. These combined multimodal findings were consistent with type IIB TAK involving the ascending aorta, arch, and descending thoracic aorta (Table 2).

**Table 2:** Numano classification of Takayasu arteritis This classification categorizes patients based on the anatomic distribution of arterial lesions:, type I involves branches of the aortic arch; types IIA and IIB include the ascending aorta and aortic arch, with IIB also affecting the descending aorta; type III involves the thoracic and abdominal aorta; type IV affects abdominal and/or renal arteries; and type V is a combination of IIB and IV (adapted from Hata et al. [6]).

Type	Description
I	Aortic arch vessels
IIA	Ascending aorta, aortic arch, and its branches
IIB	IIA vessels + descending aorta
III	Descending and abdominal aorta and/or renal artery
IV	Abdominal and/or renal arteries
V	Combination of IIB and IV

During hospitalization, the patient exhibited a superficial dermatosis in the intermammary region, clinically compatible with early-stage hidradenitis suppurativa (Hurley IA). Video electroencephalography (VEEG) indicated left hemispheric slowing with polymorphic delta activity and loss of normal background rhythms, consistent with structural cortical dysfunction. No ictal discharges or electroclinical seizure activity were recorded during monitoring. Transthoracic echocardiography revealed mild left ventricular systolic dysfunction with an aortic transvalvular gradient of 89 mmHg at the abdominal level. Systemic blood pressure was initially managed with intravenous furosemide and oral amlodipine, followed by the cautious initiation of enalapril once hemodynamic stability was achieved.

Anticoagulation with low-molecular-weight heparin was initiated during hospitalization and maintained under close clinical monitoring, without hemorrhagic complications. Immunosuppressive therapy consisted of high-dose intravenous methylprednisolone pulses for three consecutive days, followed by oral prednisone tapering and adjunctive intravenous cyclophosphamide.

Clinical stabilization and partial neurological recovery were achieved after nearly two weeks of hospitalization, with improved muscle strength and resolution of expressive aphasia. The patient was discharged after a 12-day hospital stay under multidisciplinary follow-up, including pediatric neurology, rheumatology, and cardiology services. During outpatient evaluation, she remained clinically stable, with progressive improvement in right-sided motor function, normalization of inflammatory markers, and no recurrence of neurological or systemic symptoms. Immunosuppressive therapy was continued with a favorable clinical evolution.

## Discussion

TAK is a chronic granulomatous large-vessel vasculitis affecting the aorta and its major branches. The annual incidence rate is in the range 0.4-3.4 per million individuals, with a strong female predominance [8]. Pediatric cases often follow a more aggressive inflammatory course, leading to earlier and more severe ischemic complications [9]. This case exemplifies that pattern, with a pronounced neurovascular presentation marked by supra-aortic trunk occlusion and extensive cerebral ischemia.

Clinical manifestations of TAK typically progress from nonspecific constitutional symptoms such as fever, malaise, or weight loss to overt ischemic signs, including limb claudication, syncope, hypertension, and stroke [10]. Although cerebrovascular involvement is relatively uncommon, it represents one of the most disabling complications in pediatric TAK. The acute onset in our patient, characterized by hemiparesis, global aphasia, and syncopal episodes, highlights the diagnostic challenge imposed by the disease's heterogeneous clinical spectrum [11]. Notably, the patient also presented with hidradenitis suppurativa, a chronic inflammatory skin condition involving TNF-α and IL-1β pathways. While this association with TAK is rare, recent studies indicate overlapping cytokine-mediated mechanisms that may point to a shared autoimmune background [12].

The differential diagnosis in pediatric patients presenting with large-vessel cerebral ischemia includes Moyamoya disease, primary angiitis of the central nervous system (PACNS), congenital aortic arch anomalies, fibromuscular dysplasia, and antiphospholipid antibody syndrome [13]. Unlike Moyamoya disease, which typically involves distal internal carotid stenosis with basal collaterals, our patient exhibited proximal supra-aortic occlusion and intercostal collateralization, consistent with TAK. Negative autoimmune serology and concentric wall thickening on CT angiography further supported this diagnosis over alternative vasculopathies.

Diagnosis is often delayed due to nonspecific early manifestations and the absence of reliable serological markers. In this context, cross-sectional and vascular imaging modalities, including CT angiography, magnetic resonance imaging (MRI), and positron emission tomography (PET), are essential for confirming large-vessel inflammation, defining the anatomic distribution of lesions, and assessing disease activity over time [14]. In this case, imaging findings were characteristic of advanced large-vessel involvement, with extensive supra-aortic compromise and collateral reconstitution, consistent with type IIB disease. The integration of anatomical visualization with intra-arterial pressure gradients provided critical hemodynamic insight, underscoring the complementary role of cross-sectional and functional imaging in the comprehensive assessment of pediatric TAK [15].

Management of pediatric TAK requires balancing inflammation control with preservation of organ perfusion, particularly in cases of cerebral ischemia. Early suppression of vascular inflammation remains essential to prevent irreversible vessel remodeling [7]. Corticosteroids continue to represent the cornerstone of induction therapy, often complemented by immunosuppressants such as cyclophosphamide in severe or refractory presentations. Interleukin-6 blockade with tocilizumab has emerged as an effective adjunct for sustained remission and prevention of new vascular lesions, although long-term data in pediatric cohorts remain limited [16].

In this case, the immediate therapeutic priority was stabilization of cerebral perfusion and systemic hemodynamics rather than invasive revascularization. The use of vasopressors and cautious antithrombotic therapy achieved temporary compensation while inflammation subsided [17]. Anticoagulation in large-vessel vasculitis remains controversial, as it may improve flow restoration but also carries a significant risk of hemorrhage in ischemic territories. Its use must therefore be individualized, guided by the extent of vascular occlusion and absence of intracranial bleeding [18]. Endovascular or surgical revascularization is typically reserved for the chronic or quiescent phase of TAK, once systemic inflammation and vessel wall edema have resolved. Intervening during active disease carries a high risk of restenosis, thrombosis, or procedural failure due to friable arterial walls [19]. In our patient, favorable hemodynamic stabilization and collateral compensation precluded the need for acute intervention, illustrating the importance of hemodynamic evaluation in guiding the timing of revascularization.

This case reinforces the diagnostic and therapeutic value of early multimodal vascular assessment in pediatric TAK presenting with neurovascular compromise. The integration of hemodynamic data, vascular imaging, and targeted immunosuppression is essential to optimize neurological recovery and prevent irreversible vascular injury or premature intervention. However, standardized activity scores and long-term imaging were not available to objectively assess response, and the absence of predefined endpoints limits causal interpretation. This underscores the need for further longitudinal studies to refine diagnostic criteria and therapeutic strategies in pediatric TAK with cerebral ischemia.

## Conclusions

The present case provides an illustrative example of the clinical and angiographic spectrum of pediatric TAK. The patient exhibited hallmark features, including interlimb blood pressure discrepancies, diminished upper extremity pulses, and extensive supra-aortic occlusion confirmed through multimodal vascular imaging. Hemodynamic assessment revealed a significant systolic gradient across the aortic arch, emphasizing the role of invasive evaluation in defining disease severity. The observed response to corticosteroids and cyclophosphamide underscores the importance of early immunosuppressive therapy in halting inflammatory progression, although long-term outcomes remain to be determined. The coexistence of hidradenitis suppurativa introduces a novel element of potential autoimmune overlap, highlighting the complexity of immune dysregulation in this condition. Overall, this case reinforces the diagnostic value of early imaging, the necessity of multidisciplinary management, and the need for prospective studies to better define the optimal timing of revascularization and long-term strategies for vascular and neurological preservation in pediatric TAK.

## References

[1] Gulati A, Bagga A (2010). Large vessel vasculitis. Pediatr Nephrol.

[2] Bayındır Y, Başaran Ö, Bilginer Y, Özen S (2024). Vasculitis in children. Turk Arch Pediatr.

[3] Hassold N, Dusser P, Laurent A (2024). Clinical spectrum and outcome of Takayasu's arteritis in children. Joint Bone Spine.

[4] Bolek EC, Kaya Akca U, Sari A (2021). Is Takayasu's arteritis more severe in children?. Clin Exp Rheumatol.

[5] Chitkara B, Kumar M, Singh V, Gupta A, Singh K, Khandelwal S (2025). Novel presentation of Takayasu arteritis in a young girl: severe dystonia, stroke and left ventricular thrombus. BMJ Case Rep.

[6] Hata A, Noda M, Moriwaki R, Numano F (1996). Angiographic findings of Takayasu arteritis: new classification. Int J Cardiol.

[7] Saadoun D, Garrido M, Comarmond C (2015). Th1 and Th17 cytokines drive inflammation in Takayasu arteritis. Arthritis Rheumatol.

[8] Rutter M, Bowley J, Lanyon PC, Grainge MJ, Pearce FA (2021). A systematic review and meta-analysis of the incidence rate of Takayasu arteritis. Rheumatology (Oxford).

[9] Aeschlimann FA, Twilt M, Yeung RS (2020). Childhood-onset Takayasu arteritis. Eur J Rheumatol.

[10] Misra DP, Rathore U, Kopp CR, Patro P, Agarwal V, Sharma A (2022). Presentation and clinical course of pediatric-onset versus adult-onset Takayasu arteritis-a systematic review and meta-analysis. Clin Rheumatol.

[11] Fan L, Zhang H, Cai J (2019). Clinical course and prognostic factors of childhood Takayasu's arteritis: over 15-year comprehensive analysis of 101 patients. Arthritis Res Ther.

[12] Alavi A, Shavit E, Archer J, Pagnoux C (2019). Hidradenitis suppurativa and vasculitis: a case series and literature review of a rare association. SAGE Open Med Case Rep.

[13] Keser G, Aksu K (2019). Diagnosis and differential diagnosis of large-vessel vasculitides. Rheumatol Int.

[14] Sönmez HE, Demir F, Özdel S (2021). Neuroimaging of children with Takayasu arteritis. J Child Neurol.

[15] Zhu Y, Xu XY, Mason J, Mirsadraee S (2023). Irregular anatomical features can alter hemodynamics in Takayasu arteritis. JVS Vasc Sci.

[16] Sener S, Basaran O, Kaya Akca U (2022). Treatment of childhood-onset Takayasu arteritis: switching between anti-TNF and anti-IL-6 agents. Rheumatology (Oxford).

[17] Kihara H, Uchi T, Konno S, Takenaka S, Kameda H (2023). Comprehensive management of Takayasu arteritis using immunologic and antithrombotic interventions with cerebral circulation support: a case report. Cureus.

[18] Wang J, Li C, Zheng Y (2022). Multiple aneurysms of the subclavian-axillary, abdominal aortoiliac, lower extremity, and coronary arteries in a pediatric patient of Takayasu arteritis. Ann Vasc Surg.

[19] Lim RW, Keh YS, Yeo KK, Khanna NN (2018). Takayasu's arteritis: a review of the literature and the role of endovascular treatment. AsiaIntervention.

